# Mpox mRNA-1769 vaccine inhibits orthopoxvirus replication at intranasal, intrarectal, and cutaneous sites of inoculation

**DOI:** 10.1038/s41541-024-01052-2

**Published:** 2024-12-24

**Authors:** Catherine A. Cotter, Maxinne A. Ignacio, Jeffrey L. Americo, Patricia L. Earl, Eric M. Mucker, Tiffany R. Frey, Andrea Carfi, Jay W. Hooper, Alec W. Freyn, Bernard Moss

**Affiliations:** 1 Laboratory of Viral Diseases, National Institute of Allergy and Infectious Diseases, National Institutes of Health, Bethesda, MD USA; 2 United States Army Medical Research Institute of Infectious Diseases, Fort Detrick, MD USA; 3 Moderna Inc., Cambridge, MA USA

**Keywords:** RNA vaccines, Preclinical research

## Abstract

We previously reported that mice immunized twice with a lipid nanoparticle vaccine comprising four monkeypox viral mRNAs raised neutralizing antibodies and antigen-specific T cells and were protected against a lethal intranasal challenge with vaccinia virus (VACV). Here we demonstrated that the mRNA vaccine also protects mice against intranasal and intraperitoneal infections with monkeypox virus and bioluminescence imaging showed that vaccination greatly reduces or prevents VACV replication and spread from intranasal, rectal, and dermal inoculation sites. A single vaccination provided considerable protection that was enhanced by boosting for at least 4 months. Protection was related to the amount of mRNA inoculated, which correlated with neutralizing antibody levels. Furthermore, immunocompetent and immunodeficient mice lacking mature B and T cells that received serum from mRNA-immunized macaques before or after VACV challenge were protected. These findings provide insights into the mechanism and extent of mRNA vaccine-induced protection of orthopoxviruses and support clinical testing.

## Introduction

The increased incidence of mpox in Africa and the recent global outbreak highlight the need for effective vaccines and therapeutics. Monkeypox virus (MPXV) and variola virus (VARV), which are responsible for human mpox and smallpox, respectively, belong to the orthopoxvirus genus within the chordopoxvirus subfamily of the Poxviridae^1^. The conservation of essential genes among orthopoxviruses contributes to the cross-protection of smallpox vaccines derived from vaccinia virus (VACV), the prototype orthopoxvirus^2^. Jynneos, consisting of the replication-defective modified VACV Ankara (MVA), was approved for mpox and smallpox prophylaxis based on animal studies as well as immunogenicity and safety in clinical trials^3^. During the recent global outbreak of mpox, one and two vaccinations with Jynneos provided 36% to 75% and 66% to 89% effectiveness in preventing mpox, respectively^4–6^, though anti-MPXV neutralizing antibody was low^7^. Lipid nanoparticle (LNP) vaccines containing MPXV antigen-expressing mRNAs have recently been described^8–14^ and higher neutralizing antibody to MPXV and VACV compared to MVA was demonstrated in mice^8^ and non-human primates (NHPs)^14^. Two mpox mRNA vaccines are at early stages of clinical testing (NCT05995275, NCT05988203).

The development of mRNA and subunit vaccines requires an understanding of the biology of orthopoxviruses. Two related types of infectious virus particles exist: the stable mature virion (MV) thought to be responsible for spread between hosts and the enveloped virion (EV) with an additional fragile membrane enables spread within a host^15^. The protein components of the outer membranes of the two forms of virus differ and animal studies indicate that antibodies to at least one MV membrane protein and one EV membrane provide optimal protection against infection^16–19^. The Moderna mRNA-1769 vaccine used in the present study consists of four mRNAs: two that express the MPXV MV proteins M1 and A29 (homologs of VACV L1 and A27) and two that express the MPXV EV proteins A35 and B6 (homologs of VACV A33 and B5)^8^. Our initial study demonstrated that the mRNA vaccine induces antibodies in mice that neutralize VACV and MPXV and prevents VACV spread in vitro, as well as stimulates antigen-specific CD4+ and CD8+ T cells. Two intramuscular (IM) immunizations fully protected mice against weight loss and death following an intranasal (IN) infection with VACV^8^. Here we demonstrate protection against MPXV in a mouse model and protection against VACV following a single immunization and extended protection following a booster vaccination. By using a recombinant VACV expressing firefly luciferase for live animal imaging, we show that extreme weight loss and death follow the spread of virus from the intranasal (IN) site of inoculation to the chest and abdomen of control mice. mRNA immunization greatly reduced virus replication at the site of inoculation and prevented virus spread.

There is evidence that MPXV spreads by contact in humans and has been disseminating through mucosal routes linked to sexual activities. Here, we demonstrated that IM mRNA vaccination prevented VACV replication at intrarectal (IR) and cutaneous sites of inoculation in mice. The contribution of mRNA-1769-induced antibody to protection was determined by passive inoculation of non-human primate (NHP) immune serum to mice either before or after challenge. Immune serum alone conferred protection to immune-competent and immune-compromised animals and resistance to infection correlated with the neutralization titers of the serum transferred.

## Results

### Immunogenicity and protection of mice following a single vaccination

**Fig. 1:**
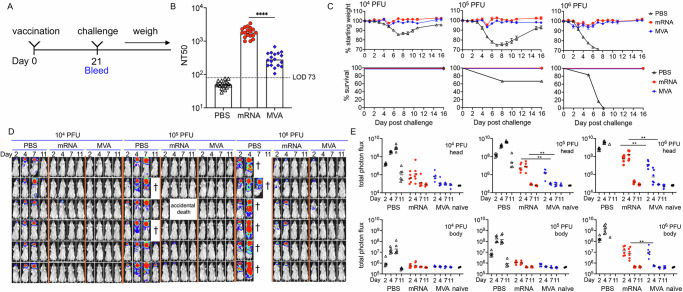
Single immunization protects against intranasal infection. **A** BALB/c mice were divided into 9 groups (*n* = 6 per group) and mock immunized with PBS or immunized with 10^7^ PFU of MVA or 8 µg of mRNA. The mice were bled on day 0 and 21 prior to IN challenge with 10^4^, 10^5^, or 10^6^ PFU of VACV WRvFire. **B** Anti-VACV MV neutralization titers expressed as NT50 of all animals receiving PBS, mRNA or MVA. Dots represent indivual animals, bars represent geometric mean titers. LOD, limit of detection. **C** Mice were weighed daily and % of starting weight and survival plotted for each group. Error bars represent standard error of the mean (SEM). **D** Luciferin was injected IP on days 2, 4, 7 and 11. Bioluminescence (BL) is depicted by a pseudocolor scale with red representing highest and blue lowest intensity. Each row represents an individual mouse imaged on successive days. **E** Photon flux was determined for head and body (chest and abdomen) region of interest (ROI). Geometric mean photon flux is indicated by bars. Significant differences between mRNA and MVA neutralization titers and photon flux for days 2 and 4 were evaluated by Mann–Whitney test. **p < 0.01, **** *p* < 0.0001.

Although many vaccines are given as a prime and boost, protection after the first dose is desirable particularly during an outbreak. To investigate protection after a single dose of the quadrivalent mRNA-1769 (referred to as mRNA hereafter), we immunized mice IM once with either mRNA or MVA for comparison and bled them 3 weeks later to determine antibody responses (Fig. 1A). Neutralizing antibodies to the M1 and A29 MV proteins are induced by the mRNAs^8^, whereas antibodies to many proteins induced by MVA may contribute to neutralization in the MV-based assay^20^. Nevertheless, the neutralizing antibody titer to MVs in the serum at three weeks after the mRNA immunization was a log higher than that achieved with MVA (Fig. 1B). The Western Reserve (WR) strain of VACV has been extensively used for studies of pathogenicity and a lethal dose 50 (LD50) of 10^5.3^ was determined for IN inoculation of DBA strain mice^21^. Here we used a VACV WR recombinant (WRvFire) that expresses firefly luciferase to enable non-invasive imaging of infected BALB/c mice^22^. The severity of infection of control BALB/c mice was related to virus dose leading to the loss of 30% or more of starting weight (required trigger for euthanasia) or death in 0 of 6 mice at 10^4^ PFU, 2 of 6 at 10^5^ PFU, and 6 of 6 at 10^6^ PFU (Fig. 1C), resulting in a calculated LD50 of 10^5.1^. In contrast, mice receiving a single injection of mRNA or MVA vaccine exhibited only minor weight loss and 100% survival even at the highest challenge dose (Fig. 1C).

Bioluminescence (BL) imaging was used to determine virus replication at the site of IN inoculation and spread in control and immunized mice following IP administration of the firefly luciferase substrate luciferin on successive days following challenge (Fig. 1D). Because firefly luciferase decays with a half-life of ~2 h in living cells^23^, BL reflects enzyme activity close to the time of analysis. Luker and Luker^24^ demonstrated a strong correlation between BL and plaque-forming units using a recombinant VACV similar to ours. In control mice challenged with 10^4^ PFU of which all survived, BL in the heads of mice reached a peak on day 7 and diminished by day 11; after challenge with 10^5^ PFU, BL was also detected in the chest by day 4 and was diminished in surviving mice by day 11; after challenge with 10^6^ PFU for which there were no survivors, BL was detected in the chest on day 2 and extended to the abdomen by day 4 (Fig. 1D). We concluded that weight loss is associated with upper respiratory infection and that severe disease coincides with spread to the lungs and abdominal organs in control mice. BL was reduced in the heads of immunized mice and few exhibited BL in the chest and none showed BL in the abdomen (Fig. 1D). However, the BL in the heads of mice immunized with mRNA and challenged with 10^6^ PFU of VACV appeared more intense on day 4 than in mice immunized with MVA.

To avoid image saturation, a relatively short exposure was used for Fig. 1D, and therefore could not reveal low levels of virus replication. Photon flux, however, provides a wide dynamic range and quantitative measurement of BL in regions of interest (ROI). Analysis of the head region confirmed high levels of virus replication in control mice at all challenge doses (Fig. 1E). The immunized mice exhibited a progressive increase in photon flux in the head with increasing challenge dose, but significantly lower than in control mice with return to baseline in all animals by day 7. Photon flux values above baseline in the bodies (which included the chest and abdomen) of immunized mice were detected only with the 10^6^ PFU challenge and were transient (Fig. 1E). However, the photon flux was significantly greater in the heads of mRNA single-immunized mice than in the MVA single-immunized mice on days 2 and 4 and in the bodies on day 4. While neither MVA nor mRNA provided sterilizing protection at the IN site of inoculation after a single shot when challenged with the potentially lethal 10^6^ PFU dose of VACV, replication was greatly reduced and transient in the head and bodies.

### Relationship of the dose of a single mRNA immunization with neutralizing antibody and protection

**Fig. 2:**
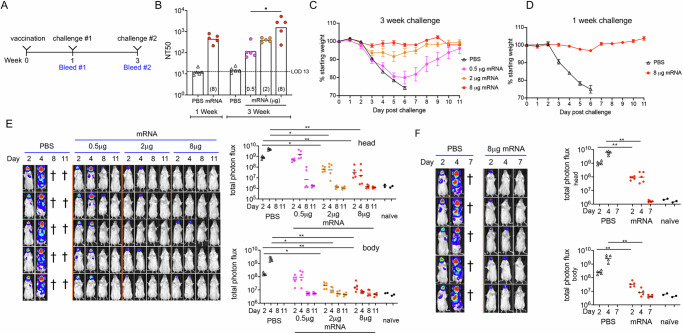
Effects of single mRNA dose and interval before challenge on protection. **A** Groups of BALB/c mice (*n* = 5) were vaccinated and challenged after 1 or 3 weeks with VACV via intranasal (IN) administration. **B** Anti-VACV MV neutralization titers expressed as NT50 determined 1 week after mock immunization with PBS or 8 µg of mRNA and 3 weeks after immunization with 0.5, 2.0, and 8 µg of mRNA. Individual animals represented by dots and geometric mean of the group represented by each bar. **C** Mice inoculated IN with 10^6^ PFU of VACV WRvFire at 3 weeks after vaccination with 0.5, 2.0, or 8.0 µg of mRNA were weighed daily and percent of starting weight plotted. Error bars represent SEM. **D** Mice inoculated IN with 10^6^ PFU of WRvFire at 1 week after vaccination with 8 µg of mRNA were weighed daily and percent of starting weight plotted. Error bars represent SEM. **E** Mice challenged at 3 weeks following vaccination were imaged following injection of luciferin on days 2, 4, 8, and 11. Bioluminescence is depicted by a pseudocolor scale with red highest and blue lowest intensity. Each row represents an individual mouse imaged on successive days. Photon flux was determined for head and body ROI for individual animals, with bar representing the geometric mean of each group. **F** Same as preceding panel except that mice vaccinated with 8 µg of mRNA and challenged after 1 week were imaged on days 2, 4, and 7. Significance for panels **B** and **E** were determined by Kruskal–Wallis test with Dunn’s post-hoc multiple comparisons and for panel **F** by Mann–Whitney test. *p < 0.05, **p < 0.01.

Groups of mice were immunized once with 0.5 µg, 2.0 µg, or 8.0 µg of mRNA. Serum was obtained at 3 weeks in each case, and also at 1 week for the 8.0 µg dose (Fig. 2A). The 3-week 50% neutralization titers (NT50) values were proportional to dose with statistical significance between the low and high doses. The titer for the 8.0 µg dose at one week was similar to the 2.0 µg dose at 3 weeks (Fig. 2B).

When challenged with 10^6^ PFU of WRvFire, the control mice rapidly lost weight and succumbed to the infection, whereas the immunized mice all survived. The mice immunized with 0.5 µg of mRNA lost about 20% of their weight when challenged at 3 weeks, while the mice receiving 2 µg of mRNA lost about 6% (Fig. 2C). Mice that received 8 µg of mRNA retained their starting weight when challenged at 3 weeks (Fig. 2C) and lost little weight when challenged at 1 week (Fig. 2D).

The mice were imaged over a period of 11 days. As in the previous experiment, the phosphate buffer saline (PBS) control mice showed high BL in the head and extensive spread of the infection to the chest and abdomen (Fig. 2E). Mice immunized with 0.5 µg mRNA exhibited strong BL in the head and some in the chest, which largely cleared by day 8 (Fig. 2E) consistent with the change in weight (Fig. 2C). Mice immunized with 2 or 8 µg of mRNA exhibited low BL in the head, and none detected in the chest or abdomen (Fig. 2E).

Photon flux measurements demonstrated significantly lower BL in the heads and bodies on days 2 and 4 of mice immunized with 2 or 8 µg of mRNA compared to the control, but the difference between 2 and 8 µg was not statistically significant (Fig. 2E). Diminution of the photon flux of some mice immunized with only 0.5 µg of mRNA was delayed and the difference from the control did not reach significance on days 2 and 4. The BL and photon flux of mice immunized with 8 µg mRNA and challenged after one week was most similar to that of mice immunized with 2 µg mRNA and challenged at 3 weeks (Fig. 2F).

In summary, three weeks after a single vaccination, NT50 values of 10^2^ to 10^3^ achieved with 2 µg or higher mRNA resulted in little or no weight loss and reduced virus replication upon IN challenge with 10^6^ PFU of VACV. A similar result was achieved 1 week after a single 8 µg mRNA vaccination. Taken together these data indicated rapid protective immunity following a single mRNA vaccination that corresponded with vaccine dose and neutralizing antibody titers.

### Increased immune response and sustained protection following a boost vaccination

**Fig. 3:**
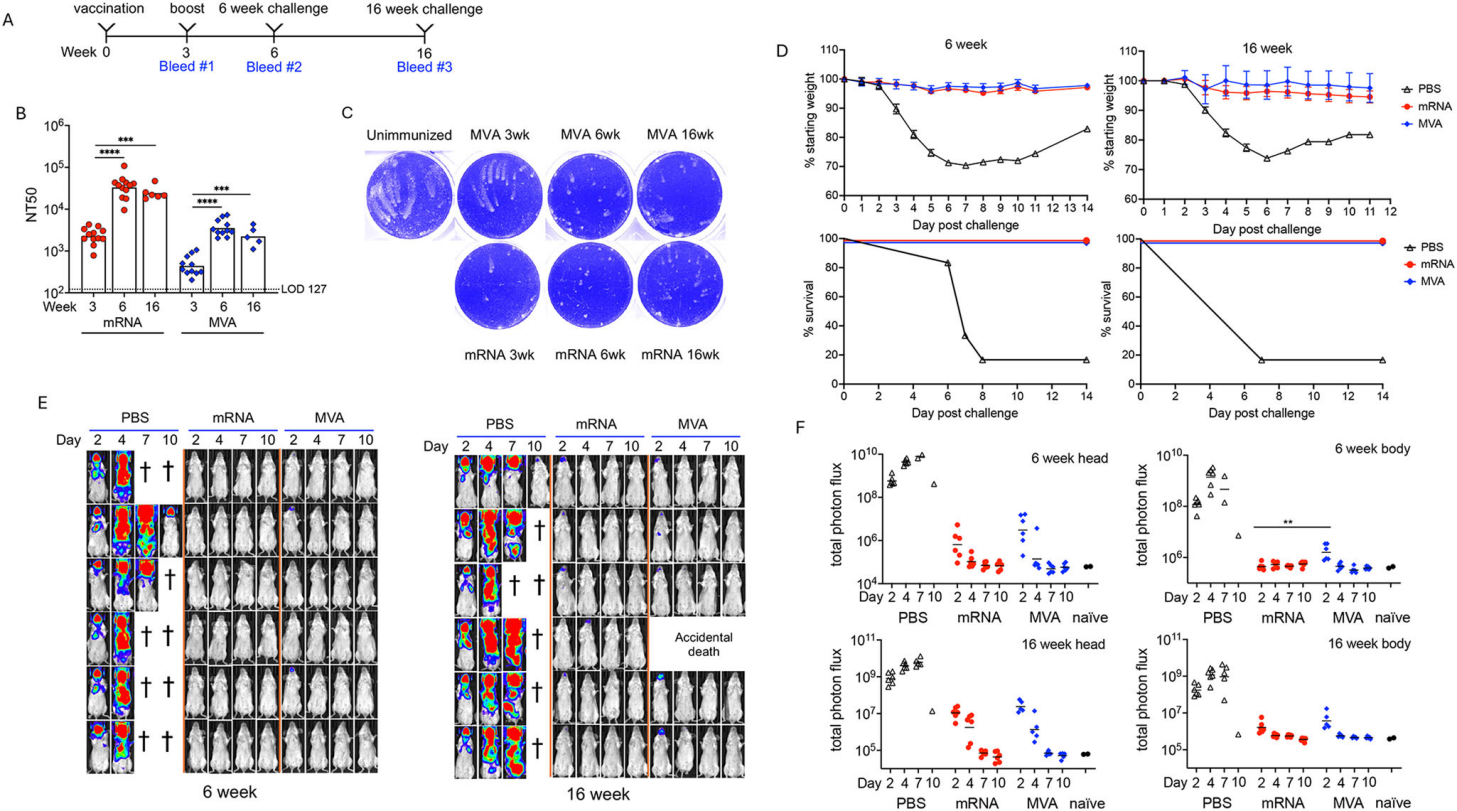
Enhanced protection after second immunization. **A** BALB/c mice were divided into 6 groups (*n* = 6) and mock immunized with PBS or immunized with 10^7^ PFU of MVA or 8 µg of mRNA on days 0 and 21 and challenged IN on weeks 3 and 16 with VACV WRvFire. **B** Anti-VACV MV neutralization titers for individual animals are expressed as NT50 with bars representing the geometric mean of each group. **C** Comet spread assay. Pooled serum diluted 1:50 was added to BS-C-1 cell monolayers at 1 h after infection with VACV strain IHD-J and stained with crystal violet after 48 h incubation at 37 °C. **D** Percent of starting weights and survival are shown as the mean of each group for each day with error bars representing SEM. **E** BL on days 2, 4, 7, and 10. Each row represents an individual mouse imaged on successive days. **F** Photon flux of head and body ROI for individual animals, with bar representing the mean of each group. Significance was evaluated by Kruskal–Wallis test with Dunn’s post-hoc multiple comparisons tests in panel (**B**) and by Mann–Whitney test in panel (**F**). **p < 0.01. ***p < 0.001, ****p < 0.0001.

To determine the impact of boosting, mice received a second inoculation of 8 µg of mRNA or 10^7^ PFU of MVA at 3 weeks after the first (Fig. 3A). For each vaccine, neutralizing antibody increased significantly after the boost and decreased slightly between 3 and 16 weeks (Fig. 3B). Again, the NT50 titers achieved with mRNA were about a log higher than with MVA.

As stated earlier, the NT50 titers refer only to neutralization of MVs. A qualitative assay to measure functional antibodies targeting the EV was conducted as follows. Antibodies to the VACV homologs of the EV proteins A35 and B6 reduce virus spread on a cell monolayer when antibody is added after virus adsorption and the plates are overlaid with liquid medium^18^. After incubation for 2 days, crystal violet staining revealed satellite plaques with a characteristic comet-like distribution in the presence of non-immune serum, which were reduced partially by 3-week serum and more completely by 6-week serum (Fig. 3C). The extent of comet inhibition decreased slightly with the serum obtained at 16 weeks (Fig. 3C).

The PBS-inoculated mice lost weight and 5 of the 6 succumbed when challenged IN with 10^6^ PFU of WRvFire, whereas mRNA- and MVA-immunized mice lost little or no weight and survived when challenged at 6 or 16 weeks (Fig. 3D). Although the MVA vaccine induced lower neutralizing antibodies than mRNA, it may have exceeded the threshold necessary for protection of mice.

Images obtained after the 6-week challenge revealed transient BL in the heads of 2 of the 6 mice that received MVA and none that received mRNA (Fig. 3E). BL was not detected in the bodies of immunized mice at week 6 (Fig. 3E). The mean photon flux levels in the heads of immunized mice were 3 and 4 logs lower than controls in MVA- and mRNA- immunized mice, respectively (Fig. 3F). Additionally, the photon flux values diminished more rapidly in the mRNA boosted mice than in mice that received only a single immunization (compare Fig. 3F and Fig. 1E). The mean photon flux in the bodies of mRNA-immunized mice were at baseline. On day 2, the photon flux in the bodies of MVA-immunized mice was significantly higher than in mRNA-immunized mice but within 2 days dropped to baseline. When challenged at 16 weeks, BL was transiently detected in the chest of one MVA-immunized mouse and a few in each group had transient BL in the head (Fig. 3E). The mean photon flux of the heads was higher than at 6 weeks but still nearly 3 logs lower than controls (Fig. 3F). The photon flux in the bodies was only slightly above baseline on day 2 before diminishing to baseline (Fig. 3F). Thus, a high degree of protection was sustained for at least 4 months.

### Protection against MPXV

**Fig. 4:**
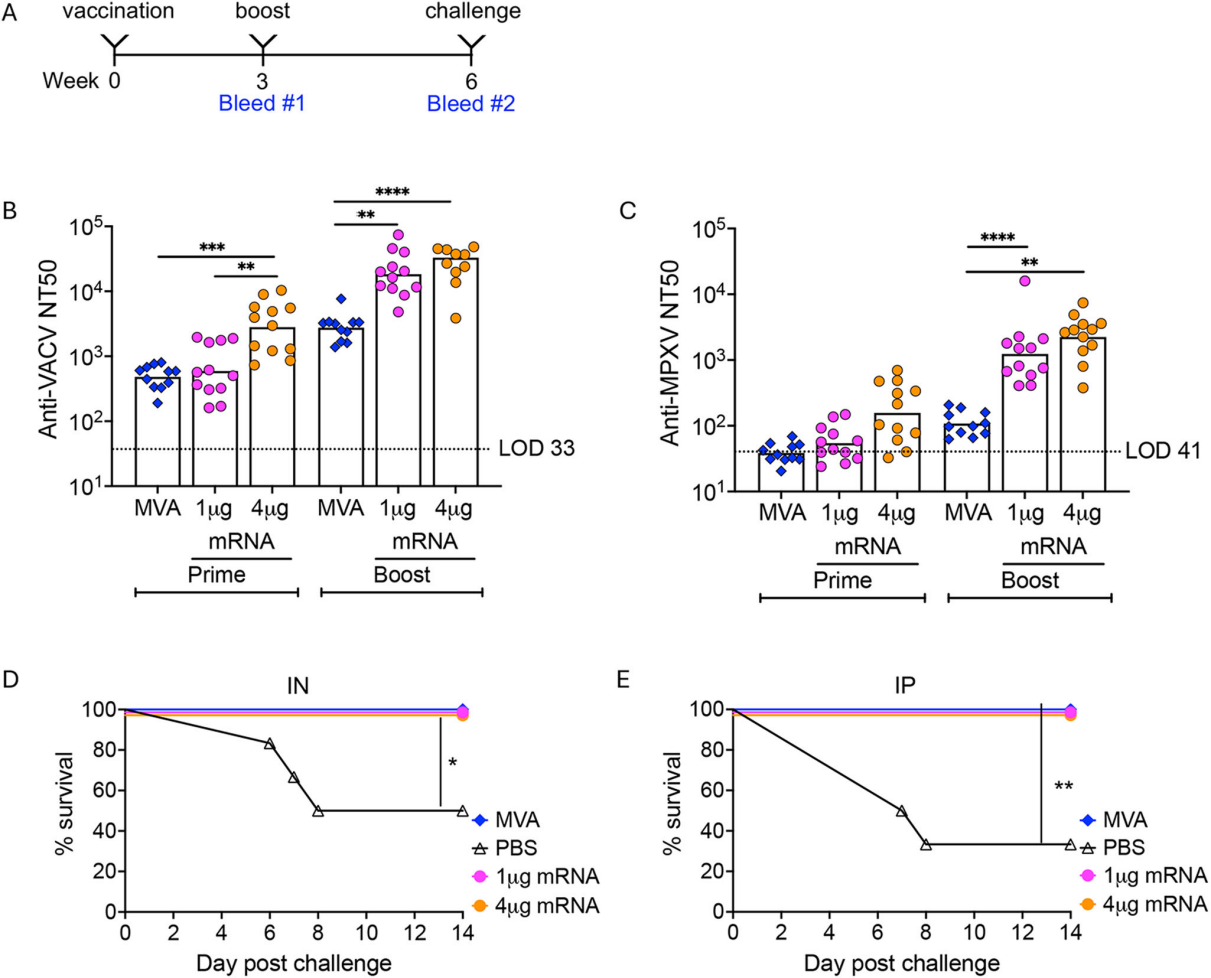
Protection of CAST mice challenged intranasally and intraperitoneally with MPXV. **A** Groups of CAST mice (*n* = 12) were primed and boosted with PBS, 10^7^ PFU of MVA, or 1 or 4 µg of mRNA before challenge IN or IP with MPXV. **B** Anti-VACV MV neutralization titers expressed as NT50 for individual animals with bars representing the geometric mean of each group. **C** Anti-MPXV MV neutralization titers expressed as NT50 for individual animals with bars representing the geometric mean of each group. **D** Six mice from each group were challenged IN with 10^5^ PFU of MPXV and survival determined as percentage of surviving mice on each day of study. **E** Six mice from each group were challenged IP with 10^4^ PFU of MPXV and survival determined as percentage of surviving mice on each day of study. Significance for panels **B** and **C** were analyzed by Kruskal–Wallis with Dunn’s post-hoc multiple comparisons tests and for panels (**D**) and (**E**) by log-rank (Mantel–Cox) survival test. *p < 0.05, **p < 0.01, ***p < 0.001, ****p < 0.0001.

Wild-derived inbred castaneous/EiJ (CAST) mice are more susceptible to MPXV and other orthopoxvirus compared to BALB/c and other common inbred mouse strains^25^. Nevertheless, CAST mice make strong IgG and T cell responses; their susceptibility to orthopoxviruses appears to be largely due to low numbers of natural killer cells^26,27^. In the next experiments, CAST mice were primed and boosted with 1 or 4 µg of mRNAs (Fig. 4A). The anti-VACV neutralizing titers of the mice primed with 4 µg of mRNA were significantly higher than the titers of mice immunized with 1 µg of mRNA or 10^7^ PFU of MVA (Fig. 4B). After boosting, the anti-VACV titers obtained with 1 and 4 µg of mRNA were both significantly higher than that obtained with MVA, but were not significantly different from each other. The anti-MPXV titers were about a log lower than the anti-VACV titers but here also the titers of mice boosted with 1 or 4 µg of mRNA were significantly higher than those that received MVA (Fig. 4C).

Previously, we determined that 5- to 7-weeks old CAST mice all succumbed to IN and IP challenges with 10^5^ and 10^4^ PFU of MPXV USA-2003, respectively^28^. In the present study, we used the same challenge doses, though the mice were 6 weeks older due to the prime and boost immunization protocol and more resistant to lethal infection. Three of the six control PBS mock-immunized mice succumbed to the 10^5^ PFU IN infection within 8 days, whereas all of the immunized mice survived for at least 2 weeks, a difference that was statistically significant (Fig. 4D). Four of the six mice challenged IP with 10^4^ PFU of MPXV succumbed while all immunized mice survived, which was also statistically significant (Fig. 4E). The similar protection with the 1 and 4 µg doses of mRNA was consistent with their similar induced neutralization titers. MVA also protected animals from lethality, even though the neutralization titers were relatively low.

### Protection against intrarectal (IR) and percutaneous infections

**Fig. 5:**
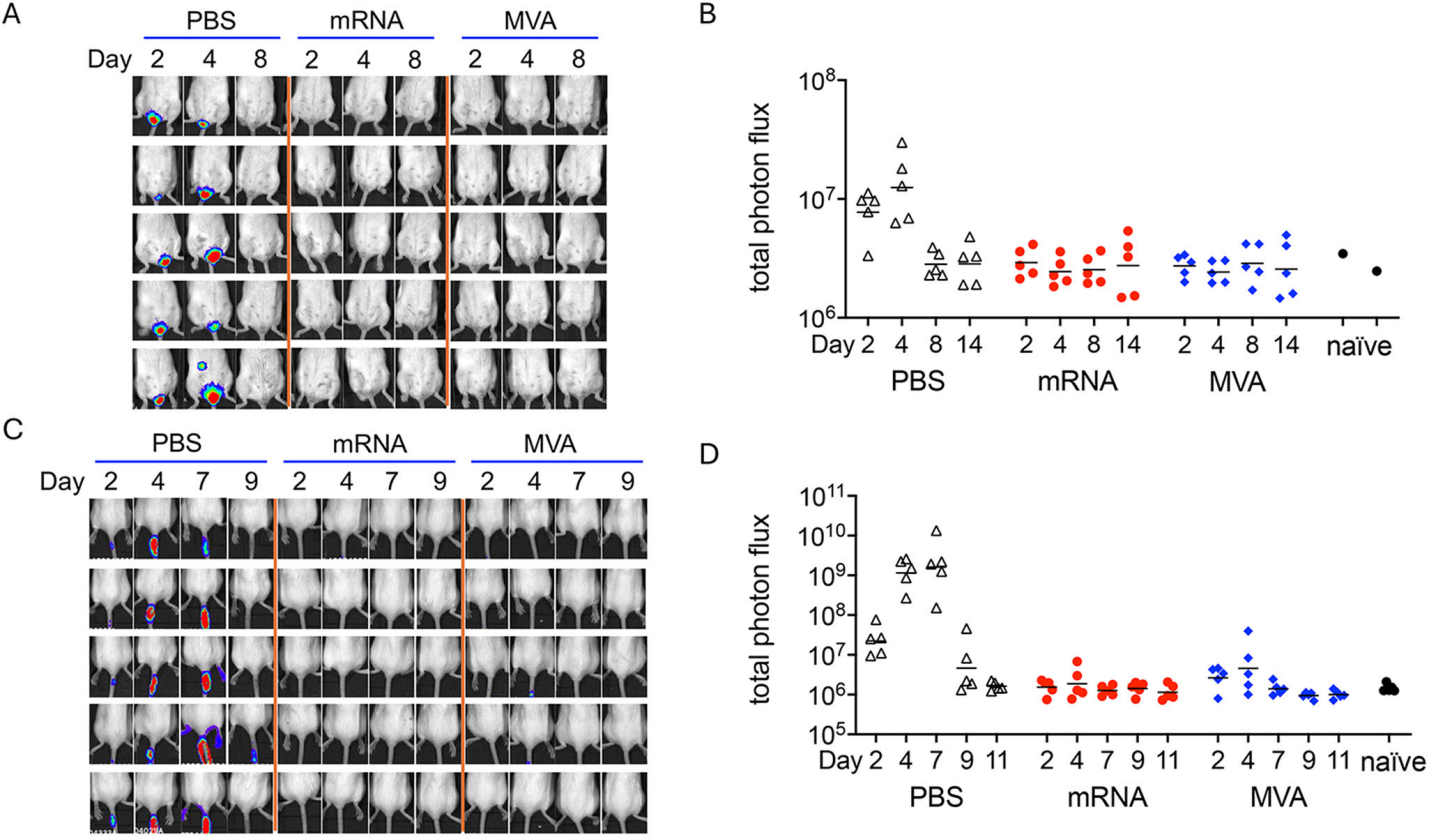
Protection from intrarectal and percutaneous infections. **A** BL of mice (*n* = 5 per group) following intrarectal infection with 10^6^ PFU of VACV WRvFire. Each row represents an individual mouse imaged on successive days. **B** Total photon flux of the rectal area for individual animals with bars representing the geometric mean of each group. **C** BL of mice (*n* = 5 per group) following percutaneous infection with 10^5^ PFU of VACV WRvFire. **D** Total photon flux of the area around the tail for individual animals with bars representing the geometric mean of each group.

Although MPXV can spread through the respiratory route, human-to-human transmission occurred mostly through male-to-male sexual activity during the recent global outbreak^29^, and lesions in the rectal mucosa were common and confined to that area in the majority of cases^30^. Additionally, there is recent evidence of sexual spread of the more pathogenic clade I MPXV in Africa^31^. It was therefore pertinent to determine whether mRNA-administered IM would protect against IR and percutaneous challenges. Mice were primed and boosted with mRNA or MVA vaccines and then inoculated with 10^6^ PFU of WRvFire IR. In control mice, local BL was detected on day 2, increased on day 4 with detectable BL in the abdominal region of one mouse, and was diminished by day 8 (Fig. 5A). In contrast, no virus replication was detected by either BL (Fig. 5A) or more sensitive photon flux measurements (Fig. 5B) in immunized mice.

The ACAM2000 smallpox vaccine was administered percutaneously in humans and caused a pustular lesion within 7 to 8 days. Here we used a similar inoculation strategy with mice except that the pathogenic VACV WRvFire was used instead of the viral vaccine strain. Following percutaneous inoculation of control mice with 10^5^ PFU of WRvFire, BL peaked on days 4 to 7 at the site of inoculation and substantially declined by day 11 (Fig. 5C, D). In contrast, no BLI or increased photon flux was detected in immunized mice. Thus, IM inoculation with either mRNA or MVA protects against IN, IR, and percutaneous infections in VACV mouse models.

### Passive immunization protects against subsequent virus infection

**Fig. 6:**
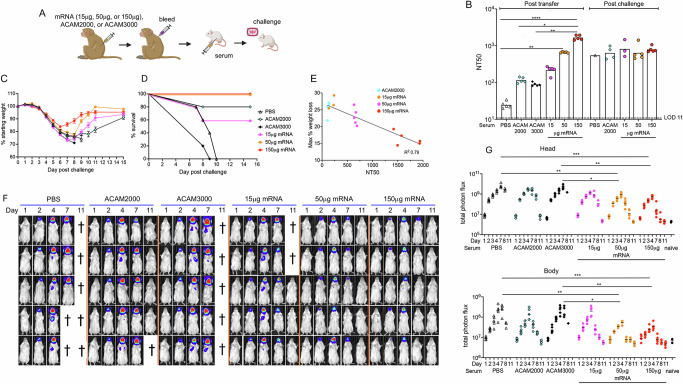
Immune serum protects against subsequent virus infection. **A** Immunization and challenge scheme. Macaques were immunized by priming and boosting with PBS, ACAM2000, ACAM3000, or 15, 50, or 150 µg of mRNA vaccine. Pooled serum was injected IP into BALB/c mice (*n* = 5 per group) and one day later the mice were challenged with 10^5^ PFU of VACV WRvFire. **B** Serum was obtained from mice 1 day post macaque serum transfer and 12 days post VACV challenge and the anti-VACV MV neutralization titer was determined for each animal with the bar representing the geometric mean of each group. **C** Mice were weighed daily following challenge and the mean of the group is reported each day with error bars representing SEM. Color key is same as in following panel. **D** Survival curves are shown for each group of mice with the percent surviving mice reported each day post challenge. **E** Relationship of weight loss to NT50 is plotted for each individual animal. *R*^2^ of 0.79 was determined by linear regression. **F** BL obtained on days indicated. Each row represents an individual mouse imaged on successive days. **G** Total photon flux was determined for head and body ROI for individual animals, with bar representing the geometric mean of each group. Signifcance was evaluated by Kruskal–Wallis test with Dunn’s post-hoc multiple comparisons tests. *p < 0.05, **p < 0.01, ***p < 0.001, ****p < 0.0001. Panel **A** was created in https://BioRender.com.

Previous studies indicated that antibody has a dominant role in protection mediated by live VACV vaccines^32–34^. It was of interest, therefore, to determine whether mice would be protected from VACV challenge by passive transfer of serum from mRNA-immunized animals. Because only small amounts of mouse serum were available, we used serum from cynomolgus macaques that was collected two weeks after priming and boosting with mRNA, ACAM2000^35^, or ACAM3000^36^ (Fig. 6A). ACAM2000 and ACAM3000 are vaccines that consist of replication-competent and replication-defective (MVA), respectively. In a preliminary experiment we determined that the titer of macaque neutralizing antibody in the blood of mice (*n* = 4) had a half-life of 7 days following IP inoculation. For the challenge experiment, mice were injected IP with pooled serum from macaques that were mock immunized with PBS or vaccinated with 15, 50, or 150 µg of mRNA or the recommended human doses of ACAM2000 or ACAM3000. The NT50 titers of the sera from mice at 1 day after injection of the macaque sera were low after transfer of ACAM2000 and ACAM3000 immune serum and only slightly higher with 15 µg mRNA but were significantly higher after transfer of the 50 and 150 µg mRNA serum (Fig. 6B). The titers of the sera at 12 days were similar to each other (Fig. 6B) due to antibody induced by the challenge virus as will be discussed below.

The mice were challenged IN with 10^5^ PFU of VACV WRvFire (1 log less than after active mRNA immunizations) at one day after IP injection of serum. Mice injected with serum from control macaques that received PBS or had been immunized with ACAM3000 rapidly lost weight and all succumbed (Fig. 6C, D). Mice that received the ACAM2000 or the 15 µg mRNA macaque serum also exhibited severe weight loss, but 3–4 of the 5 mice recovered before losing 30% of their weight. In contrast, all mice that received sera from the macaques immunized with 50 or 150 µg mRNA survived, although the former suffered more weight loss before recovering. There was an inverse relationship between the neutralization titers on day 1 and maximum weight loss of surviving mice (Fig. 6E). Nevertheless, the mice receiving macaque immune serum were not as well protected against weight loss as mice that were actively immunized even though the NT50 values were in the same range at the time of challenge. Several possible explanations for the difference are considered in the Discussion.

Mice injected with the macaque control (PBS) serum exhibited intense BL in the head and chest as did mice that received the ACAM2000 or ACAM3000 serum (Fig. 6F). Mice that received the 15 µg mRNA serum also showed strong BL in the head and detectable BL in the chest, although lower than in the mice that received non-immune serum (Fig. 6F). However, the mice that received the 50 and 150 µg mRNA sera exhibited much lower BL in the head and undetectable BL in the body. The photon flux measurements in the heads and bodies of mice that received the 50 µg and 150 µg mRNA macaque sera were significantly lower on day 7 than that of mice that received the control and ACAM3000 (MVA)-immune sera (Fig. 6G).

Although the mice receiving control serum succumbed quickly following challenge, it was possible that an immune response to the challenge virus contributed to the protection of mice receiving immune serum. To investigate this possibility, we determined the neutralizing antibody titers of the surviving mice on day 12 (Fig. 6B). The increase in NT50 was 4- to 12-fold for surviving mice that received the ACAM 2000 serum, 6- to 13-fold for the 15 µg mRNA serum and less than twofold for the 50 µg mRNA serum. The NT50 decreased for the 150 µg mRNA serum consistent with suppression of the infection and the 7-day half-life of the macaque antibodies. Thus, an immune response to the challenge virus was unlikely to benefit mice that received the 150 µg serum, though it may have helped those that received lower titer macaque serum.

### Passive immunization provides protection post-exposure to VACV infection

**Fig. 7:**
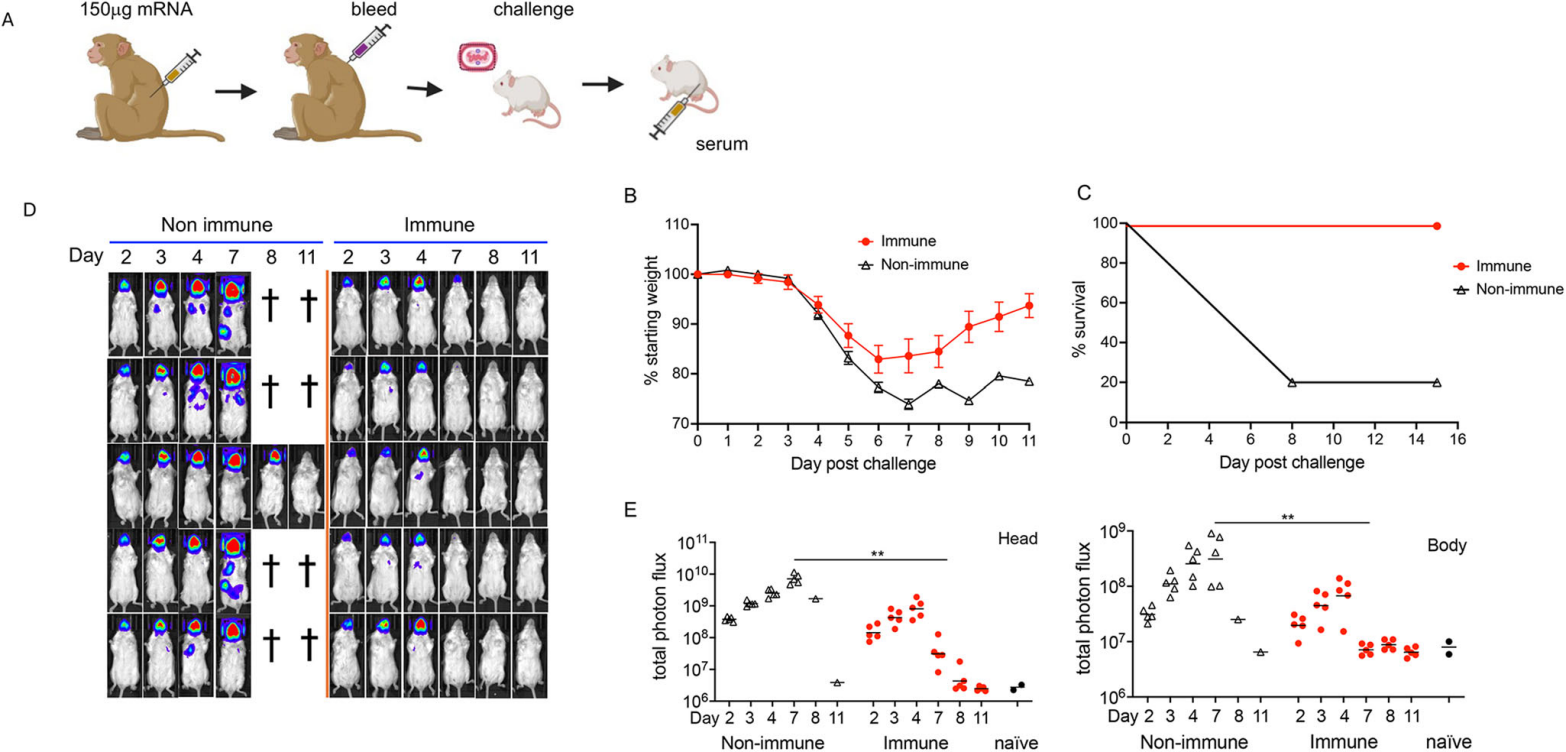
Immune serum protects against prior virus infection. **A** Immunization and challenge scheme. Macaques were immunized by priming and boosting with PBS or 150 µg of mRNA with one month between doses and peak immune serum taken two weeks post boost. Mice (*n* = 5 per group) were infected IN with 10^5^ PFU of VACV WRvFire and pooled macaque serum was inoculated IP one day later. **B** Individual mice were weighed daily following challenge with error bars representing SEM. **C** Survival curves are shown for each group of mice with the percent surviving mice reported each day post challenge. **D** BL obtained on days indicated. Each row represents an individual mouse imaged on successive days. **E** Total photon flux was determined for head and body ROI for individual animals, with bar representing the mean of each group. Significance was evaluated by Mann–Whitney test. **p < 0.01. Panel **A** was created in https://BioRender.com.

The VACV infection model uses the purified MV form of the virus for challenge. However, spread of VACV within an animal depends on the EV form of VACV with different surface proteins^37^. To determine whether the immune serum could control spread of an established infection, the same macaque serum used in Fig. 6 was added one day after the virus inoculation (Fig. 7A). Mice that received the 150 µg mRNA serum exhibited transient weight loss followed by recovery on day 7, whereas 4 of 5 mice that received nonimmune serum succumbed to the infection (Fig. 7B, C). The recovery of the immunized mice was also demonstrated by the diminution of BL in the head between day 4 and 7 (Fig. 7D). Photon flux measurements at the day 7 peak in the heads and bodies of mice that received immune serum were significantly lower compared to mice that received control serum (Fig. 7E). Taken together, passive antibody elicited by mRNA-1769 vaccination provided protection when administered before or after challenge.

### Passive immunization protects against acute disease in the absence of an adaptive immune response

**Fig. 8:**
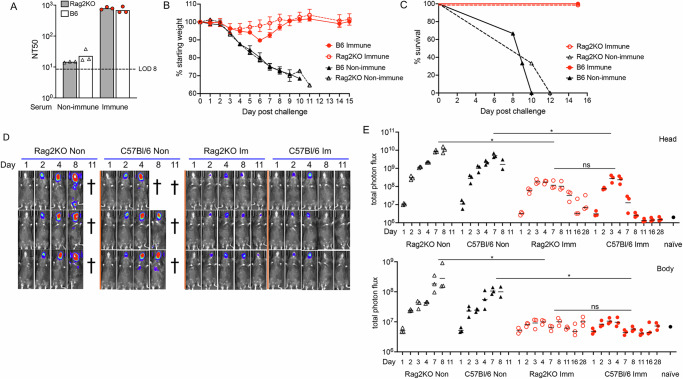
Immune serum protects immunodeficient Rag2 KO mice. **A** Anti-VACV MV neutralization titers of serum from mice (*n* = 3) at 1 day after injection of macaque serum. Bars represent the geometric mean of each group. **B** Mice were challenged one day after receiving control or immune serum and weighed daily and the mean weight of the group is reported eact day with error bars representing SEM. **C** Survival curves are shown for each group of mice with the percent surviving mice reported each day post challenge. **D** BL shown for days indicated. Each row represents an individual mouse imaged on successive days. Non, non-immune; Im, Immune. **E** Total photon flux was determined for head and body ROI for individual animals, with bar representing the mean for each group. Significance was evaluated by Kruskal–Wallis with Dunn’s post-hoc multiple comparisons tests. *p < 0.05, ns, not significant.

The recovery of weight at day 7 following virus challenge (Fig. 6C) raised the possibility of a role for an immune response elicited by the infection, although protection was shown to correlate with the abundance of neutralizing antibody transferred. To investigate a role for adaptive immunity, we passively transferred control nonimmune or 150 µg mRNA immune serum to C57Bl/6 Rag2 (recombination activating gene 2) knock-out (KO) mice, which have a deletion of the entire RAG2 protein coding region and consequently produce no mature T cells or B cells^38^. As controls, immunocompetent parental C57Bl/6 mice also received control and immune serum. The passively administered neutralizing antibody titers were similar in both mouse strains as measured 1 day post transfer (Fig. 8A). Following challenge with 10^5^ PFU of WRvFire, the C57Bl/6 and Rag2 KO mice that received the control serum rapidly lost weight and succumbed to the infection (Fig. 8B, C). By comparison, Rag2 KO and C57Bl/6 mice that received the immune serum lost relatively little weight and all survived until the experiment was ended at 28 days. A previous study found that BALB/c SCID mice passively immunized with rabbit polyclonal anti-L1, -A33 and -B5 antibodies had a 50% mean survival time of 26 days following IN challenge with VACV^18^.

Live imaging provided information regarding virus replication. BL was intense in the heads of mice that received control serum and was also detected in their chests, whereas the BL was less intense in the heads and undetectable in the bodies of Rag2 KO and C57Bl/6 mice that received immune serum (Fig. 8D). Furthermore, the mean photon flux was up to 2 logs lower in the heads of mice that received immune serum compared to nonimmune serum but there was no significant difference on combined 7 + 8 days between the Rag2 KO and C57Bl/6 mice that received the immune serum. The photon flux in the bodies of the passively immunized mice was barely above the baseline and there was no significant difference between the Rag2 KO and C57Bl/6 mice (Fig. 8E). However, virus was cleared from the head of the immunocompetent mice by day 11, whereas in the Rag2 KO mice luminescence was still detected on day 28, indicating the presence of live virus (Fig. 8E).

The conclusions of the passive transfer experiments are that antibody is sufficient to protect immunocompetent and immunodeficient mice from acute disease but that an adaptive immune response contributes to virus clearance.

## Discussion

The increasing incidence of mpox in Africa and the recent outbreak in more than 100 countries emphasize the need for vaccines that can provide rapid protection and be produced at a global scale. Furthermore, the current demographics indicate the importance of reducing human-to-human transmission particularly by sexual contact. Our previous study^8^ showed that a vaccine comprised of LNPs containing four MPXV mRNAs induce neutralizing antibodies and T cells and provide protection against weight loss and death by VACV in a murine model. Three quantitative criteria were used in the present study to assess protection following virus challenge: weight loss, survival and live BL imaging. Weight loss occurs upon infection of the upper respiratory system and may be due in part to diminished intake of liquids and food. Euthanasia is required when mice lose 30% of their starting weight so the difference between lethality and recovery of individual mice may be a fraction of a gram leading to some variability between survival of control mice in different experiments. Another variability affecting survival could be the age and weight of mice at the time of challenge, which depends on the number of vaccinations and the intervals between them in different experiments. Live BL imaging of VACV was previously shown to correlate with VACV plaque-forming units^24^ and provides a non-invasive method to assess virus replication. The three criteria gave consistent indicators of vaccine protection. The key results were: (I) mRNA vaccination protected CAST mice against IN and IP challenges with MPXV; (II) in individual unimmunized mice spread of VACV from the IN site of inoculation to the chest and abdominal organs on successive days correlated with severe disease and mortality; (III) one or two IM mRNA vaccinations greatly reduced VACV replication at the IN site and prevented systemic spread; (IV) MV neutralizing antibody was detected on day 7, increased at 3 weeks, and persisted for at least 16 weeks after vaccination; (V) MV neutralization titers and protection against VACV were mRNA dose-related; (VI) replication of VACV was local following IR and cutaneous inoculation of control mice and was undetectable at IR and cutaneous inoculation sites of vaccinated mice; (VII) passive immune serum administered to immunocompetent mice before or after IN challenge provided complete protection against a potentially lethal VACV infection; and (VIII) RAG2 KO mice unable to make an adaptive immune response were protected by passive antibody against acute disease, though they were unable to completely clear VACV from the IN site of virus inoculation by 28 days.

BL imaging was previously used to investigate the role of innate immunity on VACV replication^26,27,39^ and protection against lethal disease by Dryvax smallpox vaccine and VACV immunoglobulin^40^. Here, we used BL to determine the effect of an mRNA vaccine on replication and spread of VACV from diverse sites of inoculation. We tested escalating amounts of mRNA and found that at 2 µg or higher, a single mRNA vaccination prevented weight loss and death following IN challenge of mice. Animal imaging revealed that vaccination reduced replication by 99% as determined by photon flux measurements at the IN site of inoculation. Moreover, spread to the body was undetected after challenge with a 50% lethal dose and photon flux was reduced by more than 99% with 10X lethal dose. After a second vaccination, protection persisted for at least 4 months.

Although MPXV can be transmitted through the respiratory route, human-to-human spread of the virulent clade I virus in the Democratic Republic of the Congo is thought to have occurred predominantly by close contact in families^41,42^ and more recently through sexual activities^31^. The latter was also the predominant mechanism of spread of clade IIb MPXV during the recent global outbreak^29^. We found that following IR inoculation of control mice with VACV, replication at the site of administration was detected by luminescence on day 2, peaked on day 4, and was eliminated by day 8 without significant systemic spread, analogous to the predominantly local anogenital lesions in the current mpox outbreak^30^. In contrast, no luminescence was detected in the mRNA-immunized mice indicating little or no replication.

Control mice inoculated percutaneously developed pustular lesions and exhibited strong luminescence that peaked on day 7. However, no lesions formed in immunized mice and BL was below detection. An mRNA vaccine was also shown to reduce BL of a vaccine strain of VACV administered subcutaneously^11^. Thus, mRNA vaccination administered IM is able to protect mice against mucosal and cutaneous infections, the predominant modes of human-to-human transmission of MPXV. While we did not directly measure virus transmission in mice, reduced virus replication is likely to reduce transmission.

Although we previously demonstrated stimulation of antigen-specific T cells as well as antibodies following mRNA immunization, antibodies are generally thought to be most important for vaccine-mediated protection against VACV^32–34^. Complete protection of mice against weight loss occurred with MV NT50 titers of 10^2^ to 10^3^, which could be achieved within 7 days following active vaccination. To investigate protection by passive transfer of antibodies, we used serum from immunized NHPs because only low amounts of mouse serum were available. As precedent, several prior studies demonstrated protection against acute bacterial and viral infections including VACV following passive transfer of monkey or human serum to mice^43–46^. For our experiment, 0.5 ml of pooled sera from cynomolgus macaques that had been primed and boosted with 15, 50, or 150 µg of mRNA or with the human-recommended doses of ACAM2000 or ACAM3000 (MVA) were inoculated IP into mice. The mean NT50 values in the sera of mice one day after inoculation were in the order ACAM3000 < ACAM2000 < 15 µg mRNA, <50 µg mRNA, <150 µg mRNA and protection to the VACV challenge was directly proportional to the antibody titers, although the differences in NT50s between ACAM3000, ACAM2000 and 15 µg of mRNA did not reach statistical significance. However, there was more virus replication and greater weight loss in mice that were passively immunized compared to those actively immunized even though the neutralizing antibody titers were in the same range at the time of challenge. The difference between active and passive immunization was not due to rapid depletion of heterologous antibody as we determined that the half-life of the macaque neutralizing antibodies in mice was 7 days, similar to that of mouse antibodies^47^. Another possibility was functional incompatibility of the primate antibodies in the mouse system. Other studies, however, have shown good binding of primate IgGs to mouse Fc gamma receptors^48^ suggesting that the macaque antibodies are functional. The stimulation of innate immunity and induction of antigen-specific B and T cells by the MPXV mRNAs^8^ likely contribute to the superiority of active versus passive immunization.

Because mice are infected with the MV form of VACV and spread also depends on the EV form^37^, we also challenged the mice by the IN route one day after IP administration of serum. Presumably, the anti-MV antibodies would be most effective when present prior to exposure to MV, and the anti-EV antibodies would be crucial after initiation of infection when spread is occurring. Nevertheless, protection was similar in both cases.

Another feature of the passive transfer experiments was progressive weight loss up to day 7 following virus challenge, followed by recovery. A similar phenomenon had been reported in mice that received rhesus macaque immune serum and challenged with ZIKA virus^44^. One possibility is that an adaptive immune response to the challenge virus kicked in by day 7, helping to eliminate the infection. Indeed, the surviving mice that were passively immunized with low titer neutralizing serum exhibited an increase in neutralizing antibodies. However, the mice that received high titer serum had decreased neutralizing titers consistent with decay due to the 7-day half-life of the macaque antibody. To further investigate whether an adaptive immune response was required for protection of passively immunized mice, we employed RAG2 KO mice that cannot make mature T or B cells. These mice were protected from weight loss and virus spread as well as immunocompetent mice indicating that an adaptive immune response was not required to protect against severe acute disease. However, innate immune responses are unimpaired in RAG2 KO mice and natural killer (NK) cells^49^ likely contributed to virus clearance. Nevertheless, clearance of the virus from the site of inoculation was delayed and incomplete in the Rag2 KO mice. We conclude that antibodies induced by mRNA are sufficient for survival of a lethal infection in the mouse model. However, the better protection afforded by active immunization with mRNA-1769 is most likely due to the induction of antigen-specific B and T cells.

Our current and previous^8^ mouse studies demonstrated induction of higher anti-VACV and anti-MPXV neutralizing antibodies to MVs by mRNA than that achieved by MVA. In addition, mRNA-generated immune serum inhibited spread of EVs better than MVA. This is despite the expression of many more proteins by MVA compared to mRNA 1769. Efforts to identify the important targets of neutralizing antibodies induced by attenuated VACV strains have been unsuccessful, leading to the consideration of low neutralizing antibodies to a multitude of proteins^20^. Despite the induction of lower neutralizing antibody titers, the protection obtained by immunization with MVA was similar to that of mRNA in mice. By contrast, in NHPs MVA induced lower neutralizing antibodies and provided inferior protection against MPXV challenge^14^. Among many factors possibly contributing to differences in MVA versus mRNA protection are dose of vaccines relative to animal species and size, non-neutralizing antibodies, and cellular immunity.

In conclusion, the rapid induction of protective antibodies by mRNA-1769, the protection at IN, mucosal, and cutaneous sites in mice and the recent demonstration of protection in NHPs support further testing of this promising vaccine.

## Materials and methods

### Biosafety

All work with infectious and potentially infectious material derived from animals was conducted in BSL-2 for VACV and in registered select agent BSL-3 and ABSL-3 laboratories for MPXV by trained and smallpox-vaccinated investigators at the NIAID, NIH in Bethesda, MD using protocols approved by NIH Institutional Biosafety Committees.

### Mice

Female 5- to 6-weeks old BALB/c mice were purchased from Taconic Biosciences. Rag2 KO (B6.Cg-*Rag2*^*tm1.1Cgn*^/J; JAX stock #008449), C57BL/6J and CAST/EiJ were purchased form the Jackson Laboratory. Experiments and procedures were approved by the NIAID Animal Care and Use Committee according to standards set forth in the NIH guidelines, Animal Welfare Act and U.S. Federal law. Euthanasia was carried out when mice lost 30% of their starting weight using carbon dioxide inhalation followed by cervical dislocation in accordance with the American Veterinary Medical Association guidelines (2013 Report of the AVMA panel on euthanasia).

### Vaccines and challenge virus

MVA^33^ was purified by sucrose gradient centrifugation and titrated on primary chicken embryo fibroblasts. 10^7^ PFU of MVA in a volume of 50 µl was administered IM. The dose of mRNA-1769^8^ was 8 µg, unless stated otherwise, administered IM in a volume of 50 µl. VACV WRvFire^50^ purified by sucrose gradient sedimentation was inoculated IN in amounts ranging from 10^4^ to 10^6^ PFU in 20 µl of phosphate buffered saline with 0.05% bovine serum into one nostril. For IR inoculation, mice were held in a beaker for at least 20 min to allow them to defecate and were then anesthetized with isoflurane. The area around the rectum was swabbed with puritan calgiswab (Puitan Medical Products) and 10^6^ PFU of WRvFire was administered IR in a volume of 15 µl by inserting a pipette tip attached to a syringe into the rectum. The animals were held upside down for 2 min to prevent leakage of the material. Percutaneous inoculation of 10^5^ PFU in a volume of 10 µl was administered at the base of the tail by 20 skin scratches with a 25-gauge needle. MPXV-USA-2003-044 was purified and administered at a dose of 10^4^ PFU IN or 10^5^ PFU IP as decribed^51^.

### Passive serum transfer

Pooled heat-inactivated serum for passive transfer was obtained from cynomolgus macaques that had been primed and boosted with 15 µg, 50 µg, or 150 µg of mRNA-1769 or the recommended human doses of ACAM2000 estimated as 2.5–12.5 × 10^5^ PFU or ACAM3000 estimated as 10^8^ TCID50. BALB/c, C57Bl/6 and Rag2 KO mice received 0.5 ml of immune or control serum IP one day prior to or one day following IN challenge with 10^5^ PFU WRvFire. The day following serum transfer, mice were bled to determine levels of VACV neutralizing antibody. Mice were imaged and weighed over the next 2–4 weeks.

### Neutralization and comet inhibition assays

Virus neutralization assays were carried out by incubating sucrose gradient purified VACV WR that expresses GFP with serum dilutions as previously described^52^. For the comet inhibition assay, BS-C-1 monolayers in 12-well dishes were incubated with VACV strain IHD-J for 1 h at 37 °C. The medium was aspirated, and the monolayers washed with fresh medium to remove free virus. Pooled macaque serum diluted 1:50 was added and the plates were further incubated at 37 °C for 48 h and then fixed and stained with crystal violet.

### BL imaging

An IVIS 200 system (Perkin Elmer, Waltham, MA) was used to image infected animals as described^25^. Image collection times of up to 60 s and binning factors were held constant for all animals in an experiment. Photon flux (photons/s/cm^2^/sr) was determined for ROI including the head, body, and areas around rectum and percutaneous infection. Acquisition and analysis were performed with Living Image software (Perkin Elmer).

### Statistical analysis

Analysis was performed with Kruskal–Wallace and Dunn’s post-hoc multiple comparison, Mann–Whitney, and log-rank (Mantel–Cox) survival tests using Graphpad Prism version 10 as specificed in Figure legends.

## Data Availability

All data are available upon request to corresponding authors.
